# Supplementary material for: A highly conserved core bacterial microbiota with nitrogen-fixation capacity inhabits the xylem sap in maize plants
Source: Nat Commun. 2022 Jun 11;13:3361. doi: 10.1038/s41467-022-31113-w (PMC9187771; doi:10.1038/s41467-022-31113-w)
Supplement: Supplementary file 1 — Supplementary Information [file 41467_2022_31113_MOESM1_ESM.pdf]

Supplementary Information

A highly conserved core bacterial microbiota with nitrogen-fixation capacity inhabits the xylem sap in maize plants

Liyu Zhang[1,8], Meiling Zhang[1], Shuyu Huang[1], Lujun Li[2], Qiang Gao[3], Yin Wang[3], Shuiqing Zhang[4], Shaomin Huang[4], Liang Yuan[1], Yanchen Wen[1], Kailou Liu[5], Xichu Yu[5], Dongchu Li[1], Lu Zhang[1], Xinpeng Xu[1], Hailei Wei[6], Ping He[1], Wei Zhou[1], Laurent Philippot[7*] and Chao Ai[1,8*]

[1]Ministry of Agriculture Key Laboratory of Plant Nutrition and Fertiliser, Institute of Agricultural Resources and Regional Planning, Chinese Academy of Agricultural Sciences, Beijing, 100081, PR China.

[2]Hailun National Observation and Research Station of Agroecosystems, Key Laboratory of Mollisols Agroecology, Northeast Institute of Geography and Agroecology, Chinese Academy of Sciences, Harbin 150081, PR China

[3]Jilin Agricultural University, Changchun 130118, China

[4]Institute of Plant Nutrition, Resource and Environment, Henan Academy of Agricultural Sciences, 116 Garden Road, Zhengzhou 450002, China

[5]Jiangxi Institute of Red Soil, National Engineering and Technology Research Center for Red Soil Improvement, Nanchang 330046, China

[6]Key Laboratory of Microbial Resources Collection and Preservation, Ministry of Agriculture and Rural Affairs, Institute of Agricultural Resources and Regional Planning, Chinese Academy of Agricultural Sciences, Beijing, 100081, PR China

[7]Université Bourgogne Franche-Comté, INRAE, AgroSup Dijon, Agroécologie, 21000 Dijon, France

[8]These authors contributed equally: Liyu Zhang and Chao Ai

*Corresponding authors:

Chao Ai, E-mail: aichao@caas.cn.

Laurent Philippot, E-mail: laurent.philippot@inrae.fr.

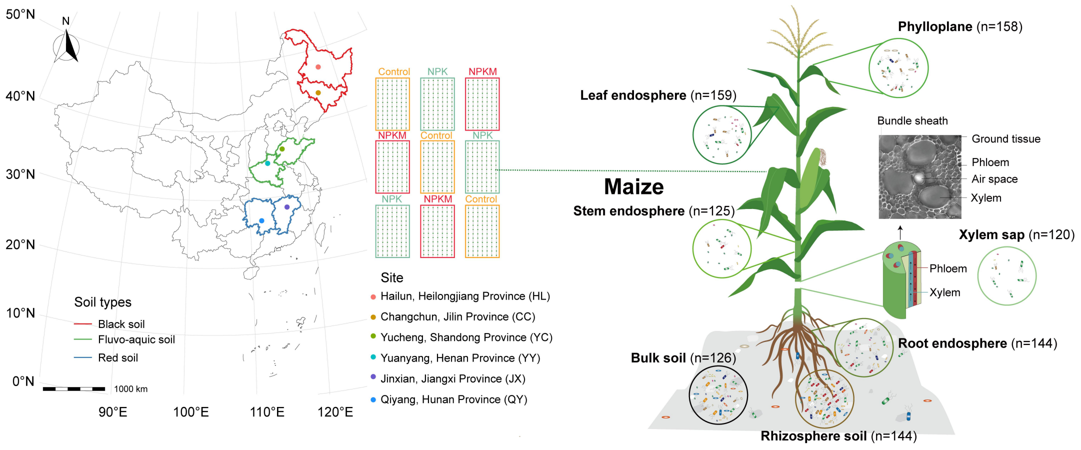

Supplementary Figure 1 Sample collection information. Geographical location of six field experiment sites in China, diagram of fertilisation treatments, and diagram of plant showing compartments from which samples were collected. Three treatments, i.e., no nitrogen fertiliser (Control), chemical fertiliser nitrogen, phosphorus, and potassium (NPK), and organic manure plus chemical fertiliser (NPKM), have been applied at each field site in triplicate plots for 29 years or more.

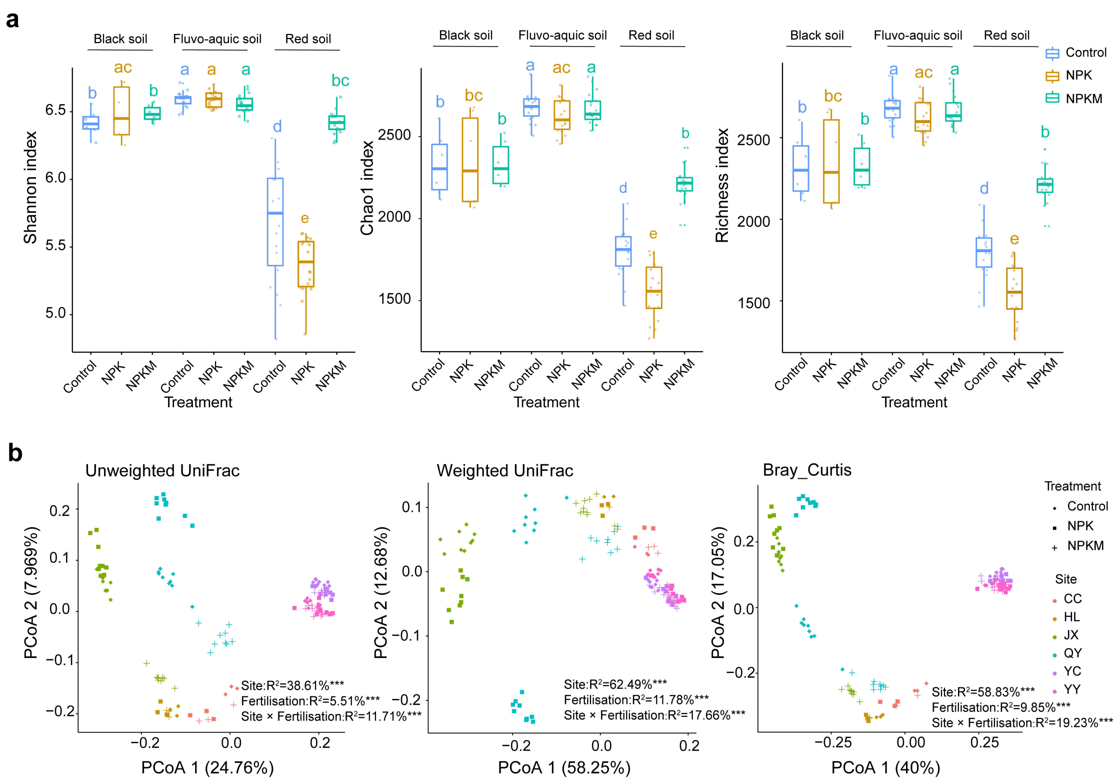

Supplementary Figure 2 Soil bacterial diversity and community structure in different soils. a, Shannon's index, Chao1 index, and richness index of bacterial microbiome in bulk soil. Numbers of replicates of samples are as follows: black soil, Control (n=3), NPK (n=3), NPKM (n=3); fluvo-aquic soil, Control; (n=9), NPK (n=9), NPKM (n=9); red soil, Control (n=9), NPK (n=9), NPKM (n=9). Horizontal bars within boxes denote medians. Tops and bottoms of boxes represent 25th and 75th percentiles, and lines extend to 1.5× interquartile range. Letters indicate statistical significance among groups using two-sided Wilcoxon test (adjusted P < 0.05 by Benjamini and Hochberg method). **b,** Unconstrained principal coordinates analysis (PCoA) with unweighted unifrac distance, weighted unifrac distance, and Bray–Curtis distance showing a significant associations of bulk soil bacterial community composition with site and fertilisation treatment. Statistical analysis was performed using permutational multivariate analysis of variance (PerMANOVA) through the "adonis" function. P values are indicated by *, i.e., *** represents P < 0.001. Source data and exact P values are provided in the Source Data file.

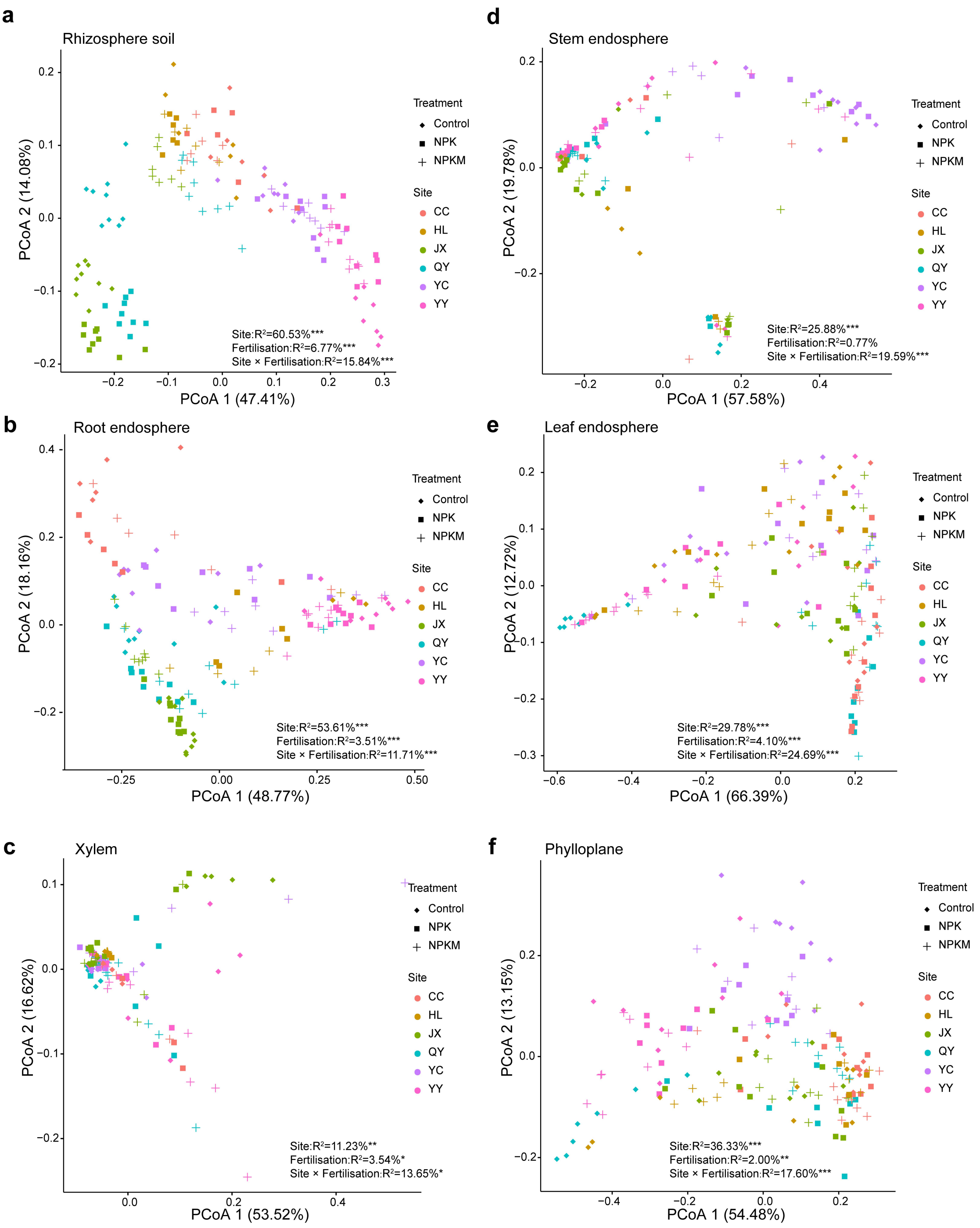

Supplementary Figure 3 PCoA analysis performed on soil and plant compartments. PCoA analysis (weighted unifrac distance) of bacterial community composition in soil (**a**), root endosphere (**b**), xylem (**c**), stem endosphere (**d**), leaf endosphere (**e**) and phylloplane (**f**). Statistical analysis was performed using permutational multivariate analysis of variance (PerMANOVA) through the "adonis" function. *P* values are indicated by *, i.e., * represents $P < 0.05$, ** represents $P < 0.01$, *** represents $P < 0.001$. Source data and exact *P* values are provided in the Source Data file.

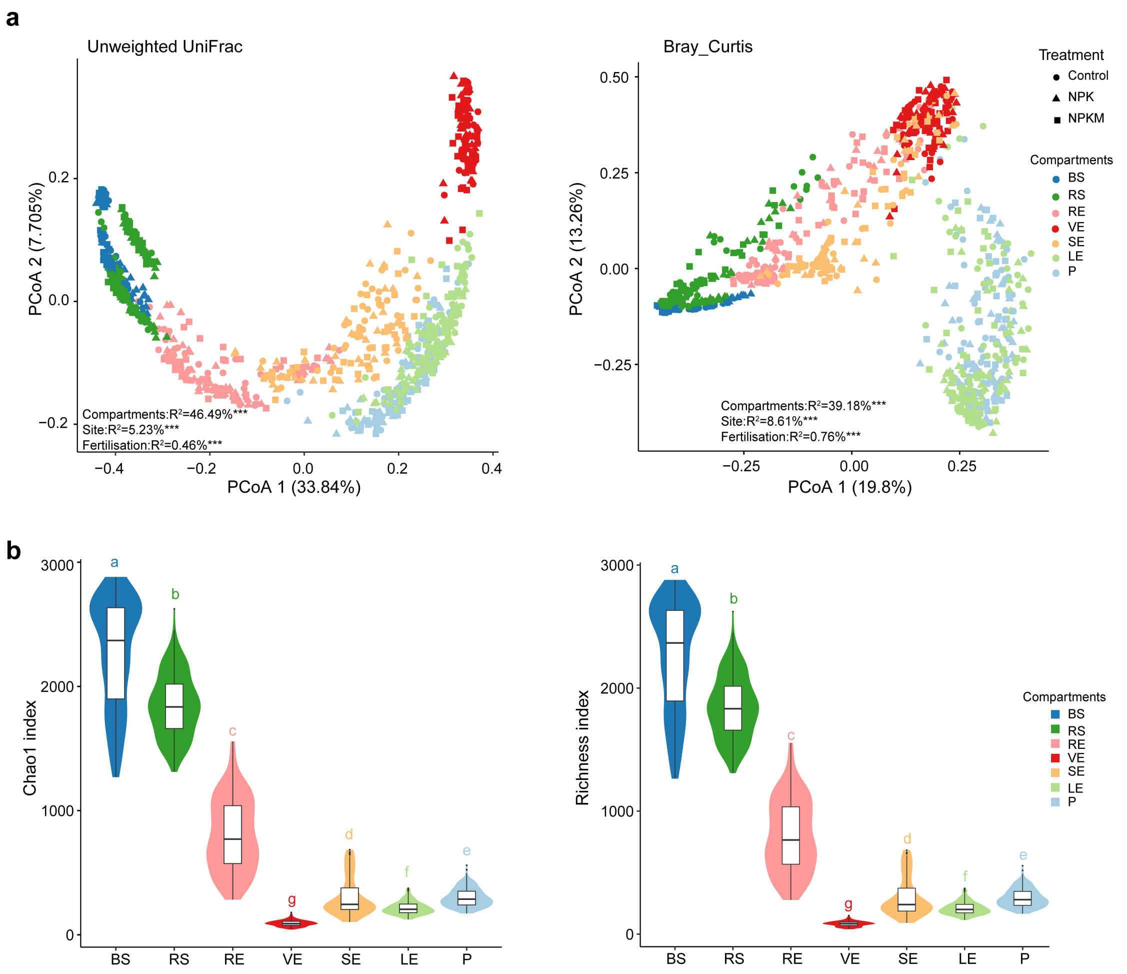

Supplementary Figure 4 Bacterial diversity in soil and plant compartments. a, PCoA analysis with unweighted unifrac distance and Bray–Curtis distance across the whole dataset showing significant associations of bacterial community composition with, in order of importance, compartment, site, and fertilisation treatment. Statistical analysis was performed using permutational multivariate analysis of variance (PerMANOVA) through the "adonis" function. *P* values are indicated by *, i.e., *** represents $P < 0.001$. **b**, Violin plot showing distributions for Chao1 index and richness index of the bacterial community in soil and plant compartments. Horizontal bars within boxes denote medians. Tops and bottoms of boxes represent 25th and 75th percentiles, and lines extend to 1.5× interquartile range. Letters indicate statistical significance among groups using two-sided Wilcoxon test (adjusted $P < 0.05$ by Benjamini and Hochberg method). The sample sizes are as follows: BS, 126; RS, 141; RE, 138; VE, 119; SE, 120; LE, 158; P, 152. Source data and exact *P* values are provided in the Source Data file.

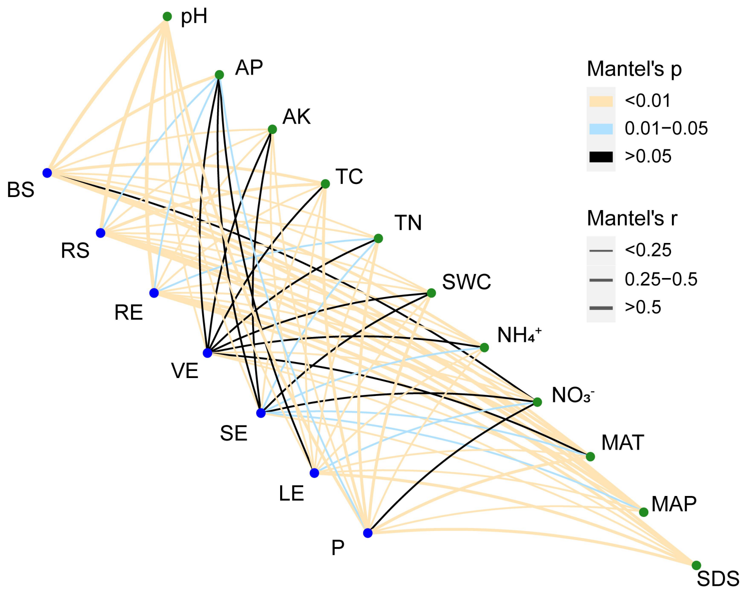

Supplementary Figure 5 Correlations between bacterial community in each compartment and environmental variables. The statistical significance of comparisons is assessed using Mantel tests based on Pearson's product moment correlation using 9999 permutations. Environmental variables: soil pH (pH); available phosphorus (AP); available potassium (AK); total carbon (TC); total nitrogen (TN); soil water content (SWC); ammonia nitrogen (NH_4^+); nitrate nitrogen (NO_3^-); mean annual temperature (MAT); mean annual precipitation (MAP); sunshine duration seasonality (SDS). Source data are provided in the Source Data file.

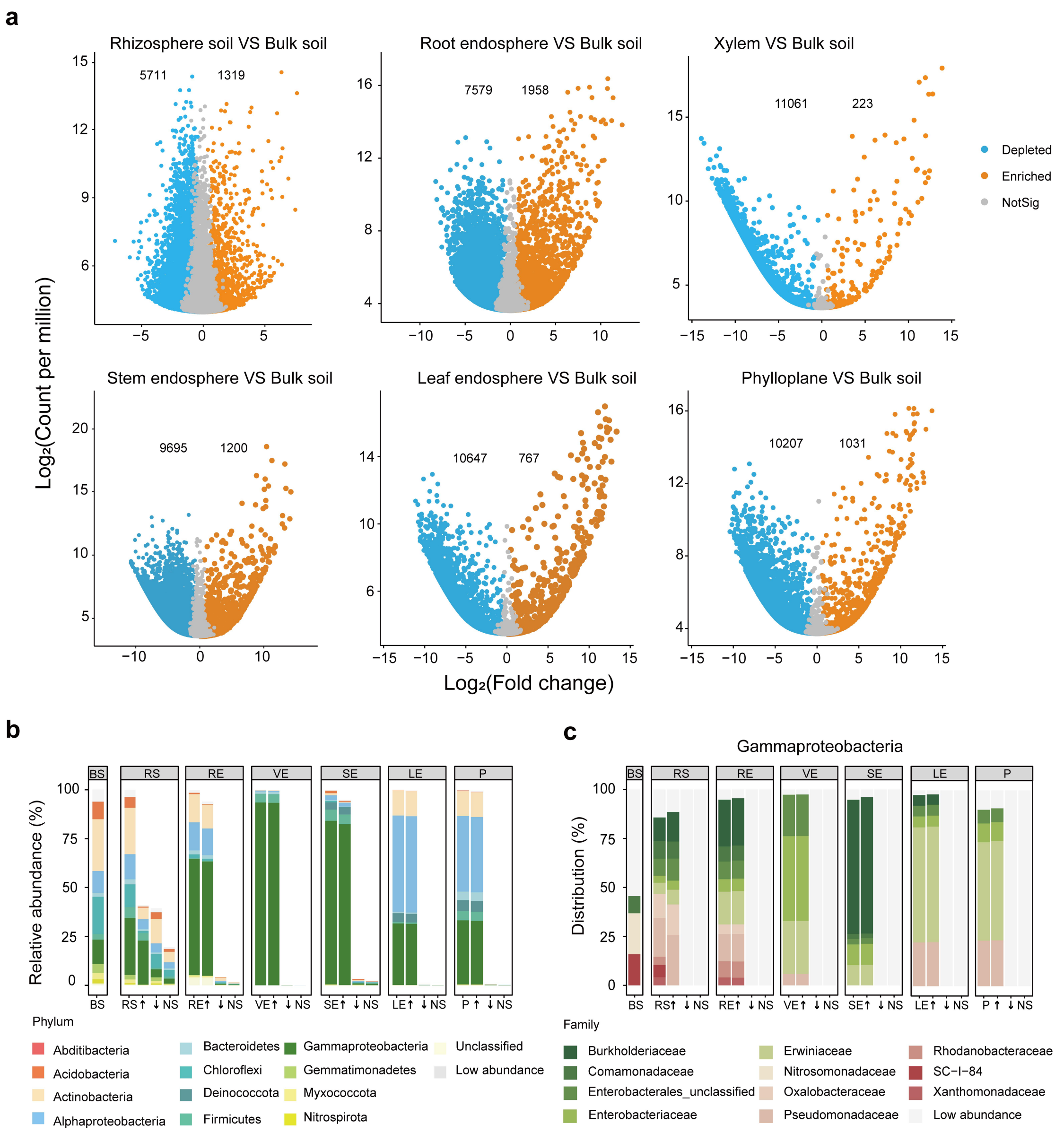

Supplementary Figure 6 Differential abundance analysis to identify OTUs associated with separation of bacterial communities between plant compartments and bulk soil. a, Volcano plots showing enriched and depleted OTUs in individual plant compartments relative to bulk soil. X axis represents the differential abundance profiles with the fold-change in a $\log_2$ scale, y axis represents average OTU abundance (as counts per million, CPM). **b,** Relative abundance of enriched OTUs (↑), depleted OTUs (↓) and not significantly different OTUs (NS) at the phylum/class level. **c,** Family-level distribution of Gammaproteobacteria. Source data are provided in the Source Data file.

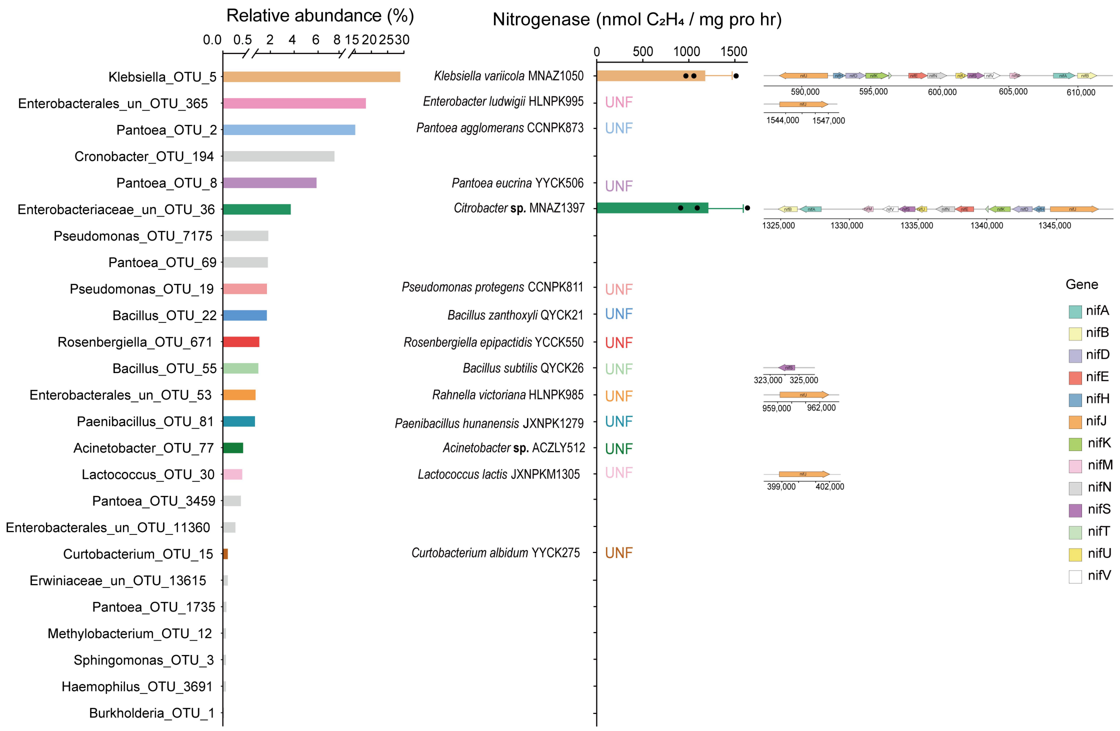

Supplementary Figure 7 Relative abundance of core OTUs and nitrogenase activity of core strains. Colored bars represent successful matches to culturable strains. Arrows mark *nif* genes in core strains, with location of each gene shown below arrows. UNF, non-N-fixers. Source data are provided in the Source Data file.

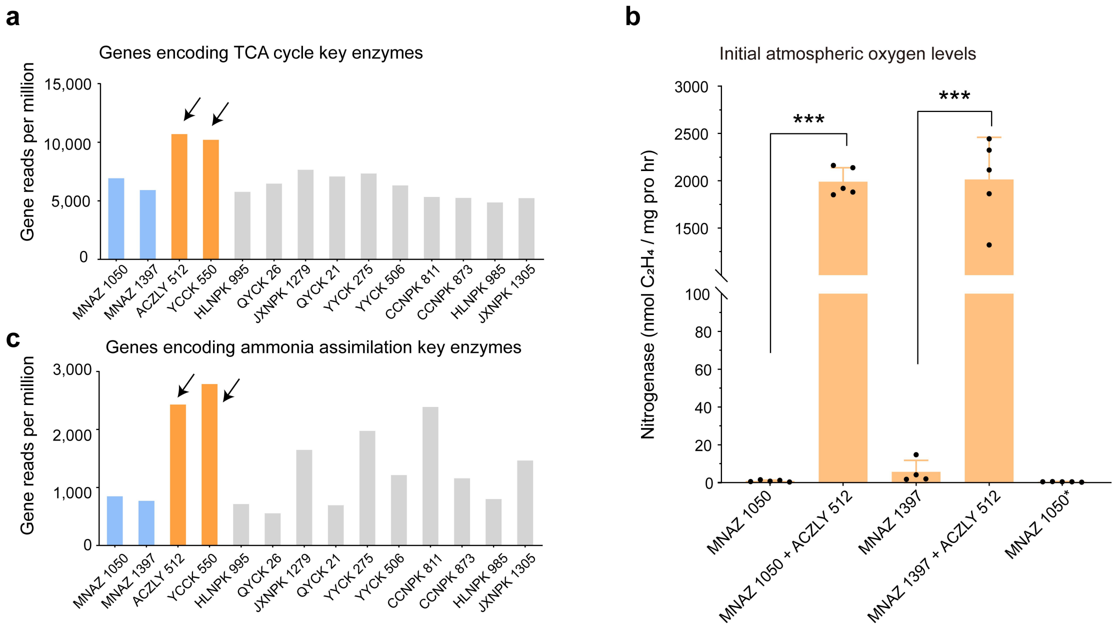

Supplementary Figure 8 Potential mechanism by which non-N-fixers assist in N-fixation. a, Genes encoding major enzymes involved in tricarboxylic acid (TCA) cycle in 14 core strains. Blue bars represent diazotrophs, orange bars represent strains that assist in N-fixation. **b**, Nitrogenase activity as determined acetylene reduction assay (ARA) under initial atmospheric oxygen levels. Number of bacteria in MNAZ1050* is equal to number of bacteria in MNAZ1050 + ACZLY512. The bars indicate mean values and the error bars indicate standard deviation (n =5). The statistical analyses were performed using a two-sided T-test. P values are indicated by *, i.e., *** represents $P < 0.001$. **c**, Genes involved in ammonia assimilation in 14 core strains. Blue bars represent diazotrophs, orange bars represent strains that assist in N-fixation. Source data are provided in the Source Data file.

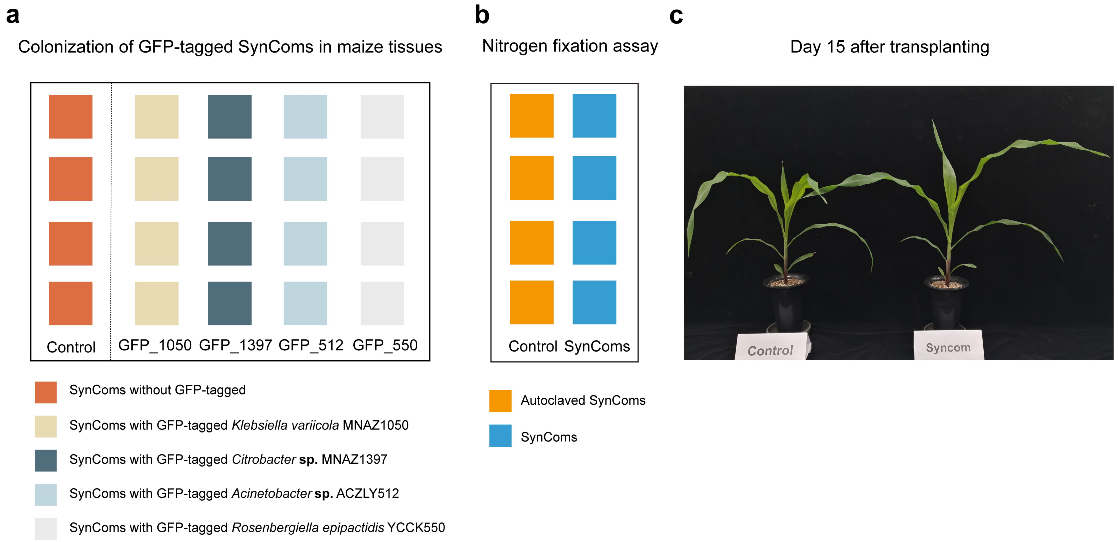

Supplementary Figure 9 Experimental design for potted plant experiments. a, Spatial distribution of experimental treatments for colonisation of GFP-tagged SynComs. Each SynCom contained one GFP-tagged strain and three other strains without GFP tags. SynComs without GFP tags served as controls. **b**, Spatial distribution of experimental treatments for N-fixation assay. Growth medium was supplemented with ^{15}N-labeled $(NH_4)_2SO_4$ instead of N fertiliser **c**, Plant growth at 15 days after transplanting.

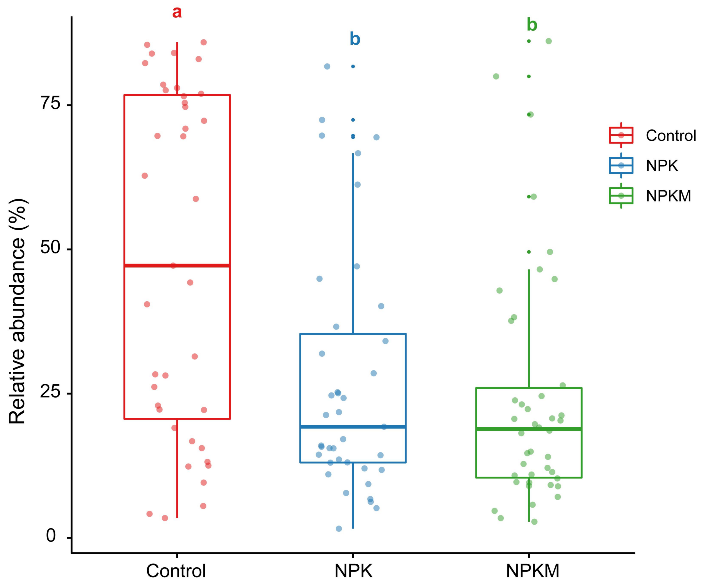

Supplementary Figure 10 Relative abundance of N-fixing bacterial strains across different fertilization regimes. The data based on the relative abundance of OTUs corresponding to N-fixing bacterial strains in the SynComs. Upper and lower whiskers extend to data no more than 1.5× the interquartile range from upper and lower edge of the box, respectively. Letters indicate statistical significance among groups using two-sided Wilcoxon test (adjusted $P < 0.05$ by Benjamini and Hochberg method). The sample sizes are as follows: Control 39; NPK, 39; NPKM, 42. Source data and exact P values are provided in the Source Data file.

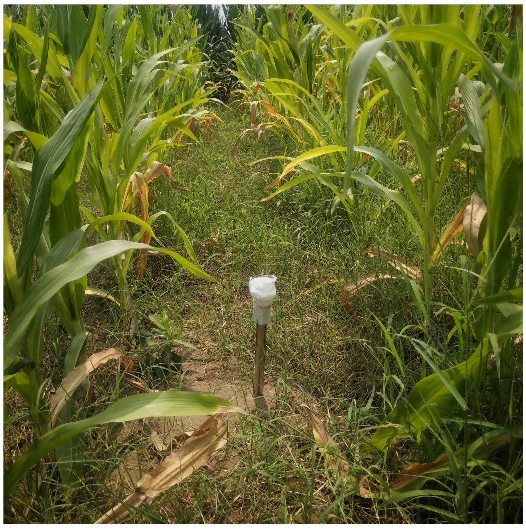

Supplementary Figure 11 A steel stick into the soil to simulate the collection of xylem sap during the field operations. Steel tube (sterilised, 2 cm in diameter, 30 cm in length) inserted into each subplot, with sterilised absorbent cotton and 9 mL ddH_2O in a sterilised bag placed on top. Recovered H_2O was used as a control to detect any external contamination during the collection of xylem sap.

Supplementary Table 1 Edaphic characteristics across six long-term fertilisation experiments.

	Site	Hailun, Heilongjiang (HL)			Changchun, Jilin (CC)		
		Control	NPK	NPKM	Control	NPK	NPKM
**Black soil**	pH	7.00±0.04^{a}	6.62±0.09^{b}	6.40±0.09^{c}	7.18±0.13^{a}	6.86±0.12^{a}	6.89±0.15^{a}
	Soil water content (SWC %)	32.52±1.13^{a}	34.73±1.00^{ab}	37.20±1.67^{b}	29.43±1.09^{a}	29.86±1.38^{a}	31.46±1.92^{a}
	Total nitrogen (TN g/kg)	2.90±0.10^{a}	3.02±0.08^{a}	3.41±0.11^{b}	2.00±0.08^{a}	2.15±0.07^{a}	2.43±0.10^{b}
	Total carbon (TC g/kg)	30.60±0.77^{a}	31.02±1.35^{a}	35.68±0.33^{b}	15.83±0.24^{a}	17.20±1.30^{a}	19.79±0.38^{b}
	Total organic carbon (TOC g/kg)	30.09±1.49^{a}	29.87±1.92^{a}	34.81±0.70^{b}	15.29±0.66^{a}	16.14±1.30^{a}	18.33±0.52^{b}
	Ammoniacal nitrogen (NH_4-N mg/kg)	3.41±1.39^{a}	3.19±0.32^{a}	3.67±0.18^{a}	3.49±0.07^{a}	4.78±0.30^{b}	7.25±0.10^{c}
	Nitrate nitrogen (NO_3-N mg/kg)	5.55±0.07^{a}	4.81±1.60^{a}	10.99±1.50^{b}	4.89±0.36^{a}	4.59±0.72^{a}	4.13±0.55^{a}
	Available phosphorus (AP mg/kg)	4.24±1.40^{a}	14.62±1.95^{b}	31.52±1.88^{c}	5.67±0.95^{a}	28.50±6.00^{b}	33.43±3.74^{b}
	Available potassium (AK mg/kg)	72.16±4.81^{a}	80.27±12.30^{a}	95.68±14.80^{a}	68.21±6.44^{a}	101.30±18.80^{b}	121.6±14.01^{b}
	**Site**	**Yucheng, Shandong (YC)**			**Yuanyang, Henan (YY)**		
		Control	NPK	NPKM	Control	NPK	NPKM
**Fluvo-aquic soil**	pH	9.20±0.07^{a}	8.88±0.08^{b}	8.74±0.11^{c}	8.75±0.1^{a}	8.28±0.12^{b}	8.24±0.12^{b}
	Soil water content (SWC %)	15.95±0.85^{a}	15.75±1.10^{a}	18.63±1.17^{b}	15.82±0.87^{a}	13.53±1.60^{b}	14.63±0.99^{ab}
	Total nitrogen (TN g/kg)	1.56±0.05^{a}	1.74±0.12^{b}	2.18 ±0.14^{c}	1.37±0.08^{a}	1.61±0.05^{b}	1.92±0.15^{c}
	Total carbon (TC g/kg)	16.76±0.44^{a}	17.60±0.62^{b}	22.36±0.82^{c}	13.73±0.55^{a}	15.10±0.69^{b}	17.95±0.83^{c}
	Total organic carbon (TOC g/kg)	6.38±0.44^{a}	7.97±1.00^{b}	12.81±1.12^{c}	6.05±0.78^{a}	7.17±0.34^{a}	10.45±1.21^{b}
	Ammoniacal nitrogen (NH_4-N mg/kg)	1.83±0.16^{a}	1.94±0.13^{a}	2.19±0.54^{a}	2.46±0.21^{a}	1.73±0.22^{b}	1.66±0.22^{b}
	Nitrate nitrogen (NO_3-N mg/kg)	3.53±0.25^{a}	8.57±2.70^{b}	11.98±4.67^{b}	4.27±0.52^{a}	16.36±5.27^{b}	15.35±2.03^{b}
	Available phosphorus (AP mg/kg)	3.39±1.46^{a}	10.58±5.77^{b}	52.99±12.48^{c}	3.79±1.46^{a}	15.06±4.16^{b}	37.70±4.73^{c}
	Available potassium (AK mg/kg)	27.76±2.70 ^{a}	48.68±4.83^{b}	46.13±7.93^{b}	21.32±3.58^{a}	57.53±18.0^{b}	67.37±7.44^{b}

	Site	Jinxian, Jiangxi (JX)			Qiyang, Hunan (QY)		
		Control	NPK	NPKM	Control	NPK	NPKM
**Red soil**	pH	5.52±0.11[a]	4.89±0.17[b]	5.86±0.22[c]	6.00±0.22[a]	5.34±0.48[b]	6.05±0.08[a]
	Soil water content (SWC %)	17.30±0.73[a]	17.06±1.32[a]	17.04±1.49[a]	27.09±7.28[a]	19.08±1.84[b]	24.71±1.07[a]
	Total nitrogen (TN g/kg)	1.79±0.11[a]	1.82±0.13[a]	2.10±0.08[b]	1.71±0.09[a]	2.13±0.09[b]	2.59±0.20[b]
	Total carbon (TC g/kg)	7.53±0.44[a]	8.45±0.27[b]	11.15±0.77[c]	7.29±0.73[a]	11.65±0.76[b]	15.93±0.77[c]
	Total organic carbon (TOC g/kg)	7.26±0.49[a]	8.09±0.38[b]	10.96±0.65[c]	6.94±0.70[a]	10.98±0.81[b]	15.71±1.71[c]
	Ammoniacal nitrogen (NH_4-N mg/kg)	2.87±0.17[a]	3.02±0.28[a]	6.79±2.64[b]	5.27±0.66[a]	4.57±1.05[ab]	4.30±0.50[b]
	Nitrate nitrogen (NO_3-N mg/kg)	2.25±0.13[a]	2.59±0.24[a]	5.45±1.61[b]	3.3±0.54[a]	3.50±0.68[a]	12.95±1.95[b]
	Available phosphorus (AP mg/kg)	11.47±2.45[a]	16.20±6.30[a]	211.55±16.07[b]	4.85±1.90[a]	88.69±18.35[b]	221.16±25.39[c]
	Available potassium (AK mg/kg)	40.02±8.04[a]	84.43±12.3[b]	131.50±32.17[b]	26.03±10.05[a]	81.72±10.89[b]	256.90±32.96[c]

Control: no fertiliser; NPK: chemical fertiliser nitrogen, phosphorus, and potassium; NPKM: organic manure plus chemical fertiliser. The values indicate mean±SD (black soil: n=3; fluvo-aquic soil: n=9; red soil: n=9).

Supplementary Table 2 Effects of site, fertilisation, and site × fertilisation on bacterial community structure in plant compartments and soil based on PerMANOVA.

	Site		Fertilisation		Site × Fertilisation		Explained variation (%)
	R^2 (%)	Pr (> F)	R^2 **(%)**	Pr (> F)	R^2 **(%)**	Pr (> F)	
**Based weighted unifrac distance:**							
Bulk soil	62.49	<0.001	11.78	<0.001	17.66	<0.001	91.93
Rhizosphere soil	60.53	<0.001	6.77	<0.001	15.84	<0.001	83.14
Root endosphere	53.61	<0.001	3.51	<0.001	11.71	<0.001	68.83
Xylem	11.23	<0.001	3.54	0.017	13.65	0.011	28.42
Stem endosphere	25.88	<0.001	0.77	0.593	19.59	<0.001	46.24
Leaf endosphere	29.78	<0.001	4.10	<0.001	24.69	<0.001	58.57
Phylloplane	36.33	<0.001	2.00	0.012	17.60	<0.001	55.93
**Based unweighted unifrac distance:**							
Bulk soil	38.61	<0.001	5.51	<0.001	11.71	<0.001	55.83
Rhizosphere soil	43.65	<0.001	4.11	<0.001	10.04	<0.001	57.80
Root endosphere	32.56	<0.001	3.29	<0.001	10.76	<0.001	46.61
Xylem	28.58	<0.001	1.85	0.038	8.87	<0.001	39.30
Stem endosphere	18.24	<0.001	2.06	0.010	10.16	<0.001	30.46
Leaf endosphere	21.48	<0.001	2.16	<0.001	7.52	<0.001	31.16
Phylloplane	24.77	<0.001	1.52	0.013	7.32	<0.001	33.61
**Based bray-curtis distance:**							
Bulk soil	58.83	<0.001	9.85	<0.001	19.23	<0.001	87.91
Rhizosphere soil	59.65	<0.001	6.34	<0.001	16.35	<0.001	82.34
Root endosphere	49.16	<0.001	4.63	<0.001	13.52	<0.001	67.31
Xylem	22.46	<0.001	7.02	<0.001	11.58	<0.001	41.06
Stem endosphere	20.69	<0.001	0.08	0.746	17.75	<0.001	38.52
Leaf endosphere	36.45	<0.001	3.71	<0.001	15.29	<0.001	55.45
Phylloplane	40.72	<0.001	1.94	<0.001	11.97	<0.001	54.63

Supplementary Table 3 Model parameters of distance-decay relationships of community similarity (1 - Bray–Curtis distance) among plant compartments and soils.

Compartments	Geographic distance			Edaphic distance			Climatic distance		
	Slope	R^2	*P*	Slope	R^2	*P*	Slope	R^2	*P*
Bulk soil	-7.85E-08	0.085	<0.001	-0.058	0.243	<0.001	-0.033	0.149	<0.001
Rhizosphere soil	-1.87E-08	0.008	<0.001	-0.049	0.207	<0.001	-0.018	0.053	<0.001
Root endosphere	-1.16E-08	0.004	<0.001	-0.029	0.103	<0.001	-0.011	0.028	<0.001
Xylem	**-1.91E-09**	**0.000**	**0.601**	**-0.009**	**0.008**	**<0.001**	**-0.001**	**0.000**	**0.237**
Stem endosphere	-1.22E-08	0.000	0.034	-0.016	0.011	<0.001	-0.006	0.002	0.002
Leaf endosphere	-1.27E-08	0.005	<0.001	-0.026	0.085	<0.001	-0.005	0.004	<0.001
Phylloplane	-2.53E-08	0.026	<0.001	-0.033	0.164	<0.001	-0.012	0.036	<0.001

Data denotes the least-squares linear regression across geographic distance, edaphic distance and climatic distance.

Supplementary Table 4 Correlations between bacterial community in plant compartments and soils and environmental variables.

**Environmental variables**	Bulk soil	Rhizosphere soil	Root endosphere	Xylem	Stem endosphere	Leaf endosphere	Phylloplane
Mean annual temperature (MAT)	**0.276(0.001)**	**0.262(0.001)**	**0.274(0.001)**	**0.069(0.047)**	**0.110(0.016)**	**0.236(0.001)**	**0.358(0.001)**
Mean annual precipitation (MAP)	**0.586(0.001)**	**0.623(0.001)**	**0.580(0.001)**	**0.112(0.001)**	**0.063(0.017)**	**0.232(0.001)**	**0.246(0.001)**
Sunshine duration seasonality (SDS)	**0.404(0.001)**	**0.421(0.001)**	**0.409(0.001)**	**0.102(0.001)**	**0.100(0.002)**	**0.295(0.001)**	**0.356(0.001)**
pH	**0.661(0.001)**	**0.811(0.001)**	**0.668(0.001)**	**0.124(0.001)**	**0.132(0.001)**	**0.181(0.001)**	**0.300(0.001)**
Soil water content (SWC)	**0.242(0.001)**	**0.207(0.001)**	**0.134(0.001)**	-0.014(0.611)	0.051(0.128)	**0.212(0.001)**	**0.344(0.001)**
Total nitrogen (TN)	**0.144(0.001)**	**0.122(0.001)**	**0.038(0.034)**	0.058(0.060)	**0.078(0.047)**	**0.183(0.001)**	**0.300(0.001)**
Total carbon (TC)	**0.281(0.001)**	**0.256(0.001)**	**0.183(0.001)**	0.056(0.078)	**0.141(0.004)**	**0.245(0.001)**	**0.300(0.001)**
Ammoniacal nitrogen (NH_4^+)	**0.435(0.001)**	**0.242(0.001)**	**0.182(0.001)**	0.006(0.427)	**0.1120(0.014)**	**0.147(0.001)**	**0.132(0.003)**
Nitrate nitrogen (NO_3^-)	0.040(0.114)	**0.180(0.001)**	**0.176(0.001)**	**0.101(0.014)**	-0.058(0.943)	**0.093(0.006)**	0.043(0.092)
Available phosphorus (AP)	**0.254(0.001)**	**0.073(0.010)**	**0.045(0.028)**	0.054(0.115)	-0.027(0.702)	0.070(0.056)	**0.071(0.042)**
Available potassium (AK)	**0.170(0.002)**	**0.108(0.002)**	**0.060(0.009)**	0.073(0.054)	-0.047(0.827)	**0.110(0.007)**	**0.166(0.001)**

The statistical significance of comparisons is assessed using Mantel tests based on Pearson’s product moment correlation using 9999 permutations.

Supplementary Table 5 Sequences of primers used in these experiments.

	16S rRNA amplicon sequencing of the V5-V7 region
Primer	Primer sequence 5'-3'
799F	AACMGGATTAGATACCCKG
1193R	ACGTCATCCCCACCTTCC
	**Quantitative PCR assays**
Primer	Primer sequence 5'-3'
799F	AACMGGATTAGATACCCKG
1193R	ACGTCATCCCCACCTTCC
PolF	TGCGAYCCSAARGCBGACTC
PolR	ATSGCCATCATYTCRCCGGA
mito 1345F	GTTTTTGGCCTTATCTTG
mito 1430R	AACCCCACTACGTACCACACCAC
	***nifH* PCR assays of isolates**
	Primer sequence 5'-3'
PolF	TGCGAYCCSAARGCBGACTC
PolR	ATSGCCATCATYTCRCCGGA
nifH-F	AAAGGYGGWATCGGYAARTCCACCAC
nifH-R	TTGTTSGCSGCRTACATSGCCATCAT
FGPH19	TACGGCAARGGTGGNATHG
PolR	ATSGCCATCATYTCRCCGGA
PolF	TGCGAYCCSAARGCBGACTC
AQER	GACGATGTAGATYTCCTG
