# Supplementary material for: A highly conserved core bacterial microbiota with nitrogen-fixation capacity inhabits the xylem sap in maize plants
Source: Nat Commun. 2022 Jun 11;13:3361. doi: 10.1038/s41467-022-31113-w (PMC9187771; doi:10.1038/s41467-022-31113-w)
Supplement: Supplementary file 2 — Reporting Summary [file 41467_2022_31113_MOESM2_ESM.pdf]

nature portfolio

Corresponding author(s): Chao Ai and Laurent Philippot

Last updated by author(s): May 31, 2022

Reporting Summary

Nature Portfolio wishes to improve the reproducibility of the work that we publish. This form provides structure for consistency and transparency in reporting. For further information on Nature Portfolio policies, see our Editorial Policies and the Editorial Policy Checklist.

Statistics

For all statistical analyses, confirm that the following items are present in the figure legend, table legend, main text, or Methods section.

n/a	Confirmed	
☐	☒	The exact sample size (n) for each experimental group/condition, given as a discrete number and unit of measurement
☐	☒	A statement on whether measurements were taken from distinct samples or whether the same sample was measured repeatedly
☐	☒	The statistical test(s) used AND whether they are one- or two-sided<br>*Only common tests should be described solely by name; describe more complex techniques in the Methods section.*
☒	☐	A description of all covariates tested
☐	☒	A description of any assumptions or corrections, such as tests of normality and adjustment for multiple comparisons
☐	☒	A full description of the statistical parameters including central tendency (e.g. means) or other basic estimates (e.g. regression coefficient) AND variation (e.g. standard deviation) or associated estimates of uncertainty (e.g. confidence intervals)
☐	☒	For null hypothesis testing, the test statistic (e.g. F, t, r) with confidence intervals, effect sizes, degrees of freedom and P value noted<br>*Give P values as exact values whenever suitable.*
☒	☐	For Bayesian analysis, information on the choice of priors and Markov chain Monte Carlo settings
☒	☐	For hierarchical and complex designs, identification of the appropriate level for tests and full reporting of outcomes
☐	☒	Estimates of effect sizes (e.g. Cohen's d, Pearson's r), indicating how they were calculated

Our web collection on statistics for biologists contains articles on many of the points above.

Software and code

Policy information about availability of computer code

Data collection	Illumina NovaSeq PE250; Illumina NovaSeq PE150; QuantStudio 6 Flex (Accurate Biotechnology); confocal laser scanning microscope (CLSM, Zeiss LSM 880)
Data analysis	Software packages for 16S amplicon data included FastQC v.0.10.1, QIIME1.9.1, QIIME2, USEARCH v.11.0, VSEARCH v.2.12.0, mothur and FAPROTAX v.1.2.1. Software packages for whole-genome sequencing data included SPAdes v3.13.1, FMAP v0.15 and NCBI PGAP (2021.01.11). We used R 3.5.3 and R 4.0.3 for statistical analyses, ggplot2 3.3.5, iTOL.v6.4, GraphPad Prism 8.0.0 and EVenn online tool for plotting. Scripts employed in the computation analyses are available under the following link: https://github.com/PlantNutrition/Liyu.

For manuscripts utilizing custom algorithms or software that are central to the research but not yet described in published literature, software must be made available to editors and reviewers. We strongly encourage code deposition in a community repository (e.g. GitHub). See the Nature Portfolio guidelines for submitting code & software for further information.

Data

Policy information about availability of data

All manuscripts must include a data availability statement. This statement should provide the following information, where applicable:

- Accession codes, unique identifiers, or web links for publicly available datasets
- A description of any restrictions on data availability
- For clinical datasets or third party data, please ensure that the statement adheres to our policy

Raw 16S rRNA gene amplicon sequence data have been deposited in the Sequence Read Archive under BioProject PRJNA795590 (https://www.ncbi.nlm.nih.gov/bioproject/PRJNA795590). The Whole Genome Shotgun data has been deposited at DDBJ/ENA/GenBank under the accession PRJNA798255 (https://www.ncbi.nlm.nih.gov/bioproject/PRJNA798255). The SILVA database is available at https://www.arb-silva.de. The eggNOG database is available at http://

eggnog5.embl.de/download/eggnog_5.0/. KEGG Filtered UniProt reference cluster is available at https://ftp.uniprot.org/pub/databases/uniprot/uniref/uniref90/. Source data are provided with this paper.

Field-specific reporting

Please select the one below that is the best fit for your research. If you are not sure, read the appropriate sections before making your selection.

☐ Life sciences ☐ Behavioural & social sciences ☒ Ecological, evolutionary & environmental sciences

For a reference copy of the document with all sections, see nature.com/documents/nr-reporting-summary-flat.pdf

Ecological, evolutionary & environmental sciences study design

All studies must disclose on these points even when the disclosure is negative.

Study description	In this study, by assessing the assembly and functions of maize microbiomes at six long-term (>29 years) fertilisation experimental sites across a wide range of soil types, climate zones, and genotypes, we found that maize specifically recruits a highly conserved core microbiota in the xylem sap. Using synthetic communities, we demonstrated that core microbiota contributes to the nitrogen nutrition of the plant through biological nitrogen fixation and promotes root development. These functionally important core taxa in the xylem sap may represent an untapped resource that can be exploited to increase crop productivity sustainably.
Research sample	To determine whether maize (Zea Mays L.) has core microbiomes in different plant compartments that can perform functions increasing host fitness, we investigated the bacterial communities of seven plant compartments (bulk soil, rhizosphere soil, root endosphere, xylem sap, stem endosphere, leaf endosphere, phylloplane) in maize genotypes (Kenwo 6, Liangyu 66, Zhengdan 958, Jundan 20 and Yedan 13) grown across a range of environmental conditions (i.e., different fertilisation regimes, soil types, climate zones).<br>Three fertilisation regimes include: no fertilisation, Control; chemical fertiliser N, phosphorus, and potassium, NPK; and organic manure plus chemical fertiliser, NPKM.<br>Three soil types include: black soils (Udolls or Typic Hapludoll according to USDA Soil Taxonomy, BSA), fluvo-aquic soils (Aquic Inceptisol according to USDA Soil Taxonomy, FSA) and red soils (Ultisols according to USDA Soil Taxonomy, RSA).<br>Three climate zones include: the middle temperate zone, warm temperate zone and subtropical zone of China.<br>The six long-term fertilisation field experiments were chosen to represent the main agricultural production areas in China.
Sampling strategy	Sampling was performed during the silking-maturity period of maize in 2019 and 2020, with the exact date of sampling depending on the developmental stage of plants at each location. In the fluvo-aquic and red soils, three individual maize plants were randomly selected from each subplot (a total of 27 maize plants per site). At the sites with black soils, we only collected one individual maize plant from each subplot (a total of nine maize plants per site), because these two long-term experiments have strict requirements for sampling to avoid large-scale damage to the entire test field. From each maize plant, we collected the mixed leaves, xylem sap, stem, roots, bulk soil. The mixed leaves sample consisted of the 2nd, 4th, and 6th leaves. To collect xylem sap, we cut off the stem mid-way between the 2nd and 3rd node from the base of the plant, and sterilised absorbent cotton in sterilised bags was placed on the cut end of the shoot. The stem sample consisted of the upper region between the 2nd and 3rd nodes. We shook whole roots vigorously to remove all loose soil to collect root samples. The roots and root-adhered soil particles were collected for further separation of the roots and rhizosphere soil in the laboratory. The bulk soil sample was collected from between the rows of maize plants.<br>In our study, to assess the assembly and functions of maize microbiomes across soil types, climate zones, and fertilisation regumes, our sample sizes (BS, n=126; RS, n=144; RE, n=144; VE, n=120; SE, n=125; LE, n=159; P, n= 158) have sufficient statistical power to answer the primary research question.
Data collection	Field data (latitude, longitude, soil temperature and sampling time) was recorded and collected by Liyu Zhang, Meiling Zhang, Shuyu Huang, Kailou Liu and Chao Ai. Climate data were obtained from the China Meteorological Data Network (http://data.cma.cn/). Liyu Zhang, Meiling Zhang and Shuyu Huang extracted DNAs, library preparation and sequencing. Amplicon libraries were sequenced by Illumina NovaSeq PE250 at Novogene. Liyu Zhang, Laurent Philippot and Chao Ai carried out the bioinformatic analysis. Isolation of bacteria from xylem sap and potted plant experiments were carried out at the laboratory by Liyu Zhang.
Timing and spatial scale	Sampling was performed during the silking-maturity period of maize in 2019 and 2020 with the exact date of sampling depending on the developmental stage of plants at each location. Samples were collected in Qiyang on July 18, 2019; Samples were collected in Yucheng on August 18, 2019; Samples were collected in Yuanyang on August 14, 2019; Samples were collected in Changchun on August 25, 2019; Samples were collected in Jinxian on June 14, 2020; Samples were collected in Hailun on August 25, 2020.<br>We collected maize samples across a latitudinal gradient in China from north to south, spanning three climate zones from the middle temperate zone to the subtropical zone. The two most distant sites (from Hailun to Qiyang) were more than 2,500 km apart, and the two closest test sites (from Yucheng to Yuanyang) were at least 300 km apart. These sites were chosen to represent the three main agricultural production areas in China.
Data exclusions	No data were excluded from the analysis.
Reproducibility	In the fluvo-aquic and red soil area, nine individual maize plants as replicates were selected from each treatment. In the black soil area, three individual maize plants as replicates were selected from each treatment. Meanwhile, analysis scripts are readily available to facilitate reproducibility.
Randomization	Individual maize plants were randomly selected in each treatment.

Blinding	The soil parameters in the field, and the N content and 15N enrichment of plant tissue for potted plant experiments were measured by researchers with no prior knowledge of the experimental design and group allocations.

Did the study involve field work? ☒ Yes ☐ No

Field work, collection and transport

Field conditions	The six long-term fertilisation field experiments were located across a latitudinal gradient in China from north to south, spanning three climate zones from the middle temperate zone to the subtropical zone. Further details of experimental sites are provided in Supplementary Table 1.
Location	The six long-term fertilisation field experiments were located in Hailun (47°21′, 126°50′), Changchun (43°48′, 125°24′), Yucheng (36° 49′, 116°34′), Yuanyang (35°00′, 113°41′), Jinxian (28°21′, 116°11′) and Qiyang (26°45′, 111°52′).
Access & import/export	Sample collection was permitted by Hailun Agro-ecosystem Experimental Station of Chinese Academy of Sciences, Jilin Agricultural University, Dezhou Experimental Station of Chinese Academy of Agricultural Sciences, Henan Academy of Agricultural Sciences, Jiangxi Institute of Red Soil and Red Soil Experimental Station of Chinese Academy of Agricultural Sciences. The heads of six long-term fertilisation experimental stations are co-authors of this study.
Disturbance	Site access and sampling was conducted in accordance with the six long-term fertilisation experimental stations' standard walking path to minimize site disturbance.

Reporting for specific materials, systems and methods

We require information from authors about some types of materials, experimental systems and methods used in many studies. Here, indicate whether each material, system or method listed is relevant to your study. If you are not sure if a list item applies to your research, read the appropriate section before selecting a response.

Materials & experimental systems

n/a	Involved in the study	
☒	☐	Antibodies
☒	☐	Eukaryotic cell lines
☒	☐	Palaeontology and archaeology
☒	☐	Animals and other organisms
☒	☐	Human research participants
☒	☐	Clinical data
☒	☐	Dual use research of concern

Methods

n/a	Involved in the study	
☒	☐	ChIP-seq
☒	☐	Flow cytometry
☒	☐	MRI-based neuroimaging
