# Supplementary material for: A highly conserved core bacterial microbiota with nitrogen-fixation capacity inhabits the xylem sap in maize plants
Source: Nat Commun. 2022 Jun 11;13:3361. doi: 10.1038/s41467-022-31113-w (PMC9187771; doi:10.1038/s41467-022-31113-w)
Supplement: Supplementary file 14 — Description of Additional Supplementary Files [file 41467_2022_31113_MOESM14_ESM.pdf]

Description of Additional Supplementary Files

File Name: Supplementary Data 1
Description: Details of experimental sites. Supplementary Data 1a: Basic and climatic information for experimental sites. Supplementary Data 1b: Fertilisation information for six long-term fertilisation experiments.

File Name: Supplementary Data 2
Description: Summary of sample collection.

File Name: Supplementary Data 3
Description: Detailed information for operational taxonomic units (OTUs) in plant compartments and soil. Supplementary Data 3a: Metadata of each sample in plant compartments and soil across six long-term fertilisation experiments. Supplementary Data 3b: Relative abundance and taxonomy information of OTUs in plant compartments and soil.

File Name: Supplementary Data 4
Description: OTUs shared among soil and plant compartments, and OTUs enriched in xylem, related to Fig. 2d.

File Name: Supplementary Data 5
Description: Occupancy and abundance patterns for bacterial community in xylem, related to Fig. 4a.

File Name: Supplementary Data 6
Description: Detailed information for cultivated non-redundant representative strains from xylem sap.

File Name: Supplementary Data 7
Description: The cross-reference of core OTUs and isolated strains, related to Fig. 4c.

File Name: Supplementary Data 8
Description: Overview of draft whole-genome sequencing. Supplementary Data 8a: Basic information for draft whole-genome sequencing. Supplementary Table 8b: Genes encoding major enzymes, related to Supplementary Figure. 8.

File Name: Supplementary Data 9
Description: Composition of growth media used for isolation of bacterial strains.

File Name: Supplementary Data 10
Description: Detailed information for potted plant experiment.

File Name: Supplementary Movie 1
Description: The procedure of collecting xylem sap samples in the field.
